# Female sexual dysfunction associated with idiopathic cerebellar ataxia: A case report

**DOI:** 10.1186/s12905-019-0833-z

**Published:** 2019-11-07

**Authors:** Carmen López-Sosa, Jorge Gámez-Zapata, Helena Iglesias-de-Sena, Montserrat Alonso-Sardón

**Affiliations:** 1 Department of Medicine, Faculty of Medicine, University of Salamanca, Campus Miguel de Unamuno, C/ Alfonso X El Sabio s/n, 37007 Salamanca, Spain; 2 Complexo Hospitalario Universitario A Coruña (CHUAC), A Coruña, Spain; 3 Area of Preventive Medicine and Public Health, School of Medicine, University of Salamanca, Campus Miguel de Unamuno. C/ Alfonso X El Sabio s/n, 37007 Salamanca, Spain; 4 Institute of Biomedical Research of Salamanca, Salamanca, Spain

**Keywords:** Female sexual dysfunction, Desire, Arousal, Orgasm, Idiopathic cerebellar ataxia, Short form of the changes in sexual functioning questionnaire (CSFQ-14)

## Abstract

**Background:**

Cerebellar ataxia affects individuals in reproductive age. To date, few clinical cases of cerebellar ataxia and involvement of the cerebellum in sexual response were reported. We report a case of a woman that need to restore skills related for execution of sexual activity and coordination of movements during sexual intercourse.

**Case presentation:**

We present a case of idiopathic cerebellar ataxia in a 25-year-old woman who was referred for sexual health consultation. The patient complained of sexual problems as follows: “I forgot the behaviors that I should adopt in a sexual encounter, and I know what to do only after paying attention to my movements.” The history of sexual behavior indicated that this patient presented a “romantic love” model. The Diagnostic and Statistical Manual of Mental Disorders (DSM-5) reports that this condition involves anorgasmia disorder and female sexual arousal disorder. In addition, there was a loss of automatism and coordination of movements in the pelvis and lower extremities. The patient’s condition improved with occupational and physical therapy combined with rehabilitation therapy based on cognitive behavioral criteria for sexual therapy.

**Conclusions:**

The case evolved from the romantic-affective model to a realistic model. The patient reported being comfortable during sexual intercourse and could explain her sexual needs to her partner. She managed to coordinate lower limb and pelvic movements, but did not reach an orgasm. Moreover, vaginal lubrication occurred with a time lag of 15–30 min after the end of sexual intercourse or masturbation.

## Background

Cerebellar ataxia is a syndrome characterized by dysfunction in balance and coordination. This condition is rare, and the estimated prevalence is 20 cases per 100,000 inhabitants [1].

The confirmation of cerebellar ataxia as the cause of sexual dysfunction is a challenge because few studies can serve as the basis for the clinical case presented herein and because previous studies on the human sexual response [2] found no correlation between this dysfunction and the cerebellum, which is involved in balance and coordination [3]. However, cerebellar disorders may explain the symptoms of the present case, because sexual activity requires coordination of movements.

Therefore, the objective of this study was to restore skills related to execution of sexual activity and adequate coordination of movements during sexual intercourse.

## Case presentation

A 25-year-old woman presented with sexual problems and was diagnosed with idiopathic cerebellar ataxia. An occupational therapist referred the patient for sexual health consultation. After the appointment, she was subjected to therapy to restore the ability to perform *Basic Activities of Daily Living* (BADLs) and physical therapy to alleviate motor and coordination deficits due to cerebellar ataxia.

### Medical history

- *Since the age of 12 years (2003–2006),* the patient presented with episodes of headache with migraine aura, dizziness, vertigo, and vomiting. The results of neurological examination and complementary tests (computed tomography (CT) scans, magnetic resonance imaging (MRI), and wakefulness electroencephalogram) were unremarkable. Hematomas, stroke, and other organic pathologies were discarded. Pharmacotherapy: Flunarizine, rizatriptan, and dexketoprofen trometamol.
- *At the age of 16 years (in 2007),* she suffered episodes of disconnection from the environment compatible with epileptic seizures together with loss of urine. Neurological tests (CT scans, MRI, somatosensory evoked potentials, cerebrospinal fluid (CSF) analysis, and sleep deprivation electroencephalogram) were performed, and the results indicated frequent generalized epileptiform discharges during the non-REM sleep phase, characterized by left temporal-parietal focal epileptiform discharges of poor persistence. Diagnosis: Epilepsy with complex partial seizures. Treatment: Valproic acid and follow-up in the neurology clinic, with 13 clinical evaluations between 2007 and 2012.
- *At the age of 22 years (in 2013),* the patient entered the Neurology Service because of complaints of paresthesia and weakness in the hands and reduced sensitivity in the lower limbs. The gait showed no signs of organicity. Complementary neurological tests (CT scans, MRI of the spine, CSF analysis, and neuromuscular examination) were conducted, and a diagnosis of progressive paraparesis and proprioceptive and vibratory sensitivity disorder as well as mild impairment of the left-central somesthetic pathway was made. Other results were unremarkable.
- *At the age of 23 years (in 2014),* the patient presented with heaviness and diffuse paresthesia in the lower limbs and returned to the Neurology Service for complete examination. Neurological examination: Lower-limb muscle strength was 4^+^ to 5. Osteotendinous reflexes (OTR) were hypoactive in the lower limbs and normal in the upper limbs. Tactile and algesic sensitivity was normal. Vibratory and positional sensitivity was reduced in the lower limbs and normal in the upper limbs. Cortical sensitivity was normal. Dysmetria in finger-nose maneuvers and dysdiadochokinesia were not observed. Romberg score of 3. Sensory ataxic gait was observed. Meningeal signs were absent. Complementary tests (CT scans, thoracic and lumbar MRI, somatosensory evoked potentials, electroencephalogram, and CSF analysis) were with unremarkable results. Autoimmune, nutritional, infectious, and demyelinating causes were discarded. Diagnosis: The etiology of gait ataxia was uncertain, but this condition may be correlated with acute posterior coronary syndrome. Treatment: Withdrawal of valproic acid. The patient was discharged with problems in ambulation (ambulation was accomplished with two crutches). No specific therapy was recommended and a mental health consultation was requested.
- *At the age of 24 years (in 2015),* the patient returned to the Neurology Clinic because of several episodes of urinary incontinence, limited ambulation, and a decrease in tactile sensitivity from the pubic to the foot region. An exhaustive study of the case was conducted again. Neurological examination: Palpation of Arnold’s nerve was painful bilaterally. Symmetrical OTR was 2/4 in the upper limbs and 3/4 in the lower limbs with an increase in the reflexogenic area. There was a tendency to present equinus deformity of the foot and claw fingers. Alteration of the arthrokinetic sensitivity and loss of vibratory sensitivity in the lower limbs was present. Moderate tactile agnosia. Romberg score of 3. Non-claudication of the limbs in Barré maneuvers. No detectable changes in the index tests. The results of finger-nose tests indicated severe bilateral dysmetria with left side predominance. Ataxic-spastic gait with increase of the base of support and unstable rotation was present. Walking on tips and heels was impossible. Tandem gait was unstable. Additional tests included positron emission tomography (measures brain metabolism with injection of fluorodeoxyglucose), and the results indicated a correlation between cerebellar hypometabolism and clinical symptoms. Diagnosis: Possible spinocerebellar ataxia of genetic origin. A genetic study was conducted: A family history of this disorder was not observed, and the results of common genetic tests (FXN, SCA1, SCA2, SCA3, SCA6, SCA7, SCA8, SCA12, SCA17, and DRPLA) were unremarkable. Therefore, a genetic cause of ataxia was discarded. Symptomatic drug treatment with ibuprofen and rizatriptan was prescribed, and the patient was referred to physical and occupational therapy. During that year, the symptoms improved, and the patient managed to walk with one crutch.
- *At the age of 25 years (in 2016),* the patient was referred for sexual health consultation.

#### Sexual medical interview

The patient attended a sexual health consultation with the help of one crutch. She reported feeling better in the morning, and instability fluctuated slightly. She complained of unsteady gait and gradual bending of the knees during ambulation. The clinical symptoms worsened when she talked to someone or attempted to focus on another task. No changes in sphincter control were observed at this stage. However, the patient presented lower tactile sensitivity from the pubic region to the feet and difficulties in discriminating temperature variations when showering.

Her **sexual history** started with heterosexual relationships at the age of 18 years. The patient had a single partner. She knew the risk of being pregnant and her male partner used a condom.

She defined the relationships as “*normal; however, since the onset of sexual problems, she did not know what to do, did not know how to act, and needed to observe her body to coordinate movements with feelings*” because she did not adequately perceive the stimuli in the lower half of the body.

In addition, the patient reported not having the same level of sexual arousal as before the dysfunction, and vaginal lubrication was insufficient sometimes occurring after sexual arousal.

The patient never practiced autoerotism and did not know whether she had experienced orgasm until that day because she could not make comparisons with previous experiences.

#### Diagnosis

The patient presented Idiopathic or secondary cerebellar ataxia. An affective-sexual pattern of “romantic love” [4] was added to this basic diagnosis. According to the Diagnostic and Statistical Manual of Mental Disorders (DSM-5) [5, 6], the patient presented anorgasmia [dysfunction No. 302.73 (F52.31)] and “subjective excitation” [dysfunction No. 302.72 (F52.22)] . In addition, other diagnoses included loss of automatism, loss of adequate coordination of movements in the pelvis and lower extremities during sexual activity, and oculomotor apraxia.

#### Sexual therapy

After reviewing the published evidence, non-standard treatment was found for this clinical case. For this reason, we propose a rehabilitation strategy based on sexual re-education according to occupational therapy theories [7] for re-establishment of BADLs execution combined with sexual therapy [8]. This sexual rehabilitation strategy is developed in 12 sessions distributed in 6 months (see Table 1), depending on patient availability, and its primary objective is to restore the skills related to sexual behavior and coordination of movements during sexual activity.

**Table 1:** Sexual therapy: Applicative model of sexual rehabilitation

Objectives	Sexual tasks	Time
1st. To facilitate excitability and autoeroticism	*A) Erotic-affective readings:* to facilitate excitability. *B) Autoeroticism:* to make the patient, through the excitement achieved with sexual fantasies that excite her, learn how to masturbate and achieve pleasure as well as improve her lubrication. Should try a minimum of 2–3 times a week.	2 months 4 sessions of 45 min each control advance and redirect
2nd. To coordinate autoerotic movements	*C) Mechanical aids:* we use vaginal vibrators so that the patient in a reclined position sees in a mirror what she does and relearns how to place the legs and contract the vaginal muscles as well as the pelvic movement to achieve arousal. Should perform 2 times a week.	2 months 4 sessions advance control
3rd. To maintain heterosexual activity with movement coordination	*D) Viewing of videos composed of sexual scenes of heterosexual couples:* the preference of our patient was of videos including sexual activity caresses, oral sex, and coital sex. Depends on the availability of the partner.	2 months 4 sessions advance control

Patient’s self-assessment is used to monitor the evolution of the case (the *booklet with sexual positions*) [9]. Moreover, a *Short Form of the Changes in Sexual Functioning Questionnaire* (CSFQ-14), adapted to a Spanish version [10] is applied before and after the intervention.

CSFQ-14 is a 14-item self-assessment tool that evaluates behaviors and problems in 3 phases of the sexual response cycle, including desire (items 2–6), arousal (items 7–9), and orgasm (items 11–13). Item 1 reflects pleasure and satisfaction. Items 10 and 14 are not specific to any phase of the sexual response cycle. The 14 items are rated by the patient using a 5-point Likert scale (1 = never to 5 = every day) or intensity (1 = none to 5 = much), except for items 10 and 14, for which 1 corresponds to “every day” and 5 corresponds to “never”). For all items, higher scores reflect greater sexual functioning. The total CSFQ-14 score ranges from 14 to 70 points. A total score of ≤41 suggests overall sexual dysfunction. The desire subscale score ranges from 5 to 25 (cutoff point of 15), and the arousal and orgasm subscale scores range from 3 to 15 (cutoff points of 12 and 11, respectively).

### Follow-up and outcomes

#### Before the intervention

The total CSFQ-14 score of 25 was lower than the reference value (41) and indicated overall sexual dysfunction. The scores obtained in each subscale were: desire (9), excitation (5), and orgasm (3). These values were lower than the cut-off points and were close to the lower limit of the range. The orgasm subscale presented lower scores (see Table 2).

**Table 2:** CSFQ-14 (Female Clinical version): Response Before-After Intervention (numerical values)

Items	Before	After	Subscales
1. Compared with the most enjoyable it has ever been, how enjoyable or pleasurable is your sexual life right now?	1	3	Sexual Pleasure *(Item 1)* Before – After 1 - 3
1-No enjoyment or pleasure.			
2-Little enjoyment or pleasure.			
3-Some enjoyment or pleasure.			
4-Much enjoyment or pleasure.			
5-Great enjoyment or pleasure.			
2. How frequently do you engage in sexual activity (sexual, intercourse, masturbation, etc.) now?	2	4	Sexual Desire *(Items 2–6)* Before – After 9 - 19
1-Never.			
2-Rarely (once a month or less).			
3-Sometimes (more than once a month, up to twice a week).			
4-Often (more than twice a week).			
5-Every day.			
3. How often do you desire to engage in sexual activity?	4	4	
1-Never.			
2-Rarely (once a month or less).			
3-Sometimes (more than once a month, up to twice a week).			
4-Often (more than twice a week).			
5-Every day.			
4. How frequently do you engage in sexual thoughts (thinking about sex, sexual fantasies) now?	1	4	
1-Never.			
2-Rarely (once a month or less).			
3-Sometimes (more than once a month, up to twice a week).			
4-Often (more than twice a week).			
5-Every day.			
5. Do you enjoy books, movies, music or artwork with sexual content?	1	3	
1-Never.			
2-Rarely (once a month or less).			
3-Sometimes (more than once a month, up to twice a week).			
4-Often (more than twice a week).			
5-Every day.			
6. How much pleasure or enjoyment do you get from thinking about and fantasizing about sex?	1	4	
1-No enjoyment or pleasure.			
2-Little enjoyment or pleasure.			
3-Some enjoyment or pleasure.			
4-Much enjoyment or pleasure.			
5-Great enjoyment or pleasure.			
7. How often do you become sexually aroused?	2	4	Sexual Arousal *(Items 7–9)* Before – After 5 - 11
1-Never.			
2-Rarely (once a month or less).			
3-Sometimes (more than once a month, up to twice a week).			
4-Often (more than twice a week).			
5-Every day.			
8. Are you easily aroused?	1	4	
1-Never.			
2-Rarely (much less than half the time).			
3-Sometimes (about half the time).			
4-Often (much more than half the time).			
5-Always.			
9. Do you have adequate vaginal lubrication during sexual activity?	2	3	
1-Never.			
2-Rarely (much less than half the time).			
3-Sometimes (about half the time).			
4-Often (much more than half the time).			
5-Always.			
10. How often do you become aroused and then lose interest?	2	5	
5-Never.			
4-Rarely (much less than half the time).			
3-Sometimes (about half the time).			
2-Often (much more than half the time).			
1-Always.			
11. How often do you experience an orgasm?	1	3	Sexual Orgasm *(Items 11–13)* Before – After 3 - 8
1-Never.			
2-Rarely (much less than half the time).			
3-Sometimes (about half the time).			
4-Often (much more than half the time).			
5-Always.			
12. Are you able to have an orgasm when you want to?	1	1	
1-Never.			
2-Rarely (much less than half the time).			
3-Sometimes (about half the time).			
4-Often (much more than half the time).			
5-Always.			
13. How much pleasure or enjoyment do you get from your orgasm?	1	4	
1-No enjoyment or pleasure.			
2-Little enjoyment or pleasure.			
3-Some enjoyment or pleasure.			
4-Much enjoyment or pleasure.			
5-Great enjoyment or pleasure.			
14. How often do you have painful orgasm?	5	5	
5-Never.			
4-Rarely (much less than half the time).			
3-Sometimes (about half the time).			
2-Often (much more than half the time).			
1-Always.			
TOTAL CSFQ-14 (Items 1–14)	25	51	

#### Sexual therapy

The progress of the patient for each proposed task was as follows.

##### First objective

The patient achieved a “*facilitating sexual fantasy*” that stimulated sexual arousal. The self-assessment indicated that patient’s condition was improved and confidence was increased, although the lag in objective sensation of vaginal lubrication was not decreased.

##### Second objective

Patient’s symptoms improved with use of vibrator, and she reported experiencing a sensation of suffocation that might be an orgasm. The patient used vibrator in 10 instances and reported a shortening of the time needed to reach sexual arousal. However, this mechanical aid did not please her and thus she did not want to incorporate it in the sexual relationship with her partner at this stage because she did not have much confidence and preferred to focus on her fantasies. She reported being comfortable in the man-on-top position.

##### Third objective

After viewing erotic videos, the patient reported being happier and more confident in the intimate relationship. She maintained 3 heterosexual contacts and was more comfortable and less fearful of rejection. In addition, the explanation to her partner about her sexual dysfunction helped her relax and be less expectant. She rated the sexual encounter as enjoyable, did not have the anxiety experienced before the intervention, and was able to focus on her feelings. She changed her posture, moving from the man-on-top position to the woman-on-top position, and although her legs were slightly inactive, it did not constitute a problem because her partner helped her.

#### Current status after intervention

The total CSFQ-14 score of 51 was higher than the reference value or cutoff point (41), suggesting the absence of overall sexual dysfunction. Although all subscale scores were improved, including desire (19), sexual arousal (11), and orgasm (8), only the score for desire (19) was higher than the cut-off point (15), whereas the score for sexual arousal (11) was lower than the cutoff point (12) and the score for orgasm (8) was even lower than the cutoff point (11; see Table 2).

The rehabilitation program produced positive results according to the CSFQ-14, because among other benefits, the patient was more confident during sexual activity, allowed the partner look at her during the sexualstimulation, did not experience shame or rejection for her dysfunction, participated more in the relationship, was more active, and adopted other sexual positions. By contrast, the patient complained of difficulties in performing movements and not achieving the levels of sexual arousal that she experienced before the disease onset. The confusion and embarrassment about intimacy in the lower extremities and genitalia area was lower after therapy.

The patient achieved a “*facilitating sexual fantasy*” that stimulated sexual arousal and perceived arousal both objectively (she knew that she was lubricated by introducing her fingers into the vagina) and subjectively before ataxia.

Masturbation in front of the mirror allowed her to internalize the coordination and rhythm of movements even without looking in the mirror. Erotic movies helped her remember and coordinate the sequence of sexual movements. This strategy improved her confidence during sexual activity as she informed the sexual partner about her problem, became sexually aroused, and concentrated on her performance. She changed from the man-on-top to the woman-on-top position. She did not use a vibrator with the partner at this stage.

The case evolved to a realistic, non-romantic, sexual affective model, consistent with her medical history of cerebellar ataxia. She reports: “*I want to live the moment, I do not know what will happen next, there are no fairytales*”. She was comfortable during sexual intercourse, explained her sexual needs to the partner, and coordinated movements. She believed having reached orgasm, and lubrication was consciously evident with a time lag of 15 min after the end of sexual intercourse or masturbation.

## Discussion and conclusions

The patient presented with cerebellar ataxia and signs of oculomotor apraxia of idiopathic origin. However, clinical evidence indicated the need to continue etiological diagnosis, because the patient used many medications, including valproic acid, which are controlled and present adverse effects at the mitochondrial level [2, 11–13].

The data on sexual activities provided by the patient allowed us to conclude that she presented with an affective-loving pattern known as *“romantic love model”* [4], which evolved to an *“empty love model*”, which differed from the first model by the absence of commitment and development of passion alone, as confirmed by the patient’s comment: “*The future is unpredictable and it is better to live each day*.”

We propose that anorgasmia may be of a psychological origin rather than physical, because neurophysiological and neurodiagnostic studies and functional tests conducted by Whipple and Komisaruk [14–19] found no correlation between the cerebellum and human orgasm. Therefore, we could not confirm whether anorgasmia was secondary to ataxia because the patient reported not knowing whether she had orgasms before the intervention.

Regarding the patient’s request—unexpected and unique—about “*learning what to do during sexual intercourse*”, she reported to have forgotten the sequence of sexual movements and being confused. Her description of the condition was as follows: *“What do I have to do when I perform a sexual act, I do not what how to do with my legs, ..., I need to see my legs to know what to do*”. It may indicate that this condition is due to cerebellar ataxia because of its effect on the coordination of movements. Execution of refined movements requires coordination and synchronization of many actions in different muscles. This refined control is carried out primarily by the cerebellum, which has connections with many other regions of the nervous system. The patient’s comment “*I need to observe my body to know what to do*” supports the diagnosis of oculomotor apraxia [2, 11–13, 20–24] and possibility of rehabilitation of skills using occupational and physical therapy strategies [7].

We based this study on the hypothesis that our patient had forgotten how to coordinate movements during sexual activity and execution of BADLs. Therefore, the design of a rehabilitation program that restored coordinated movements during sexual intercourse was successful. Hypometabolism diagnosed on fluorodeoxyglucose-positron emission tomography could explain the delay in vaginal lubrication.

The main limitation is that this clinical case on human sexuality involves personal psychological factors, which cannot be reproduced in an experimental animal model. It is known that the cerebellum coordinates movements; however, no experimental models have been developed for assessment of sexual behaviors to date. Therefore, it is essential to develop a model of sexual rehabilitation that can be applied to clinical cases of individuals with compromised automatism of sexual behaviors.

In summary, the patient presented with a sexual problem related to coordination of movements during sexual activity, which may be due to cerebellar ataxia combined with an anorgasmia disorder diagnosed during therapy. However, the etiology of anorgasmia was not confirmed. Moreover, there was a time lag in the perception of vaginal lubrication.

The rehabilitation program was positive because among other benefits, the patient began to feel comfortable during sexual activity, let the sexual partner look at her during sexual intercourse, was not ashamed of her dysfunction, participated more in the relationship, was more active, and adopted other sexual positions. By contrast, the patient complained of difficulties in executing movements and not being able to reach the levels of sexual arousal that she experienced before the disease onset. Her confusion and embarrassment regarding caresses of the lower extremity and genitalia were lower after therapy.

These results indicate that the patient made progress considering recovery of most sexual functions during sexual rehabilitation.

## Supplementary information

**Additional file 1.** CARE Checklist (2013) of information to include when writing a case report.

## Data Availability

Dataset generated and analyzed during the current study are available from the mains authors upon reasonable request from another investigator or institution dedicated to investigation to email addresses: lopezsosa@usal.es or sardonm@usal.es.

## Abbreviations

BADLs: Basic Activities of Daily Living
CSF: Cerebrospinal Fluid
CSFQ-14: Short Form of the Changes in Sexual Functioning Questionnaire
CT: Computed Tomography scans
DSM-5: Diagnostic and Statistical Manual of Mental Disorders
MRI: Magnetic Resonance Imaging
OTR: Osteotendinous Reflexes
