# Supplementary material for: Female sexual dysfunction associated with idiopathic cerebellar ataxia: A case report
Source: BMC Womens Health. 2019 Nov 7;19:133. doi: 10.1186/s12905-019-0833-z (PMC6839138; doi:10.1186/s12905-019-0833-z)
Supplement: Supplementary file 1 — Additional file 1. CARE Checklist (2013) of information to include when writing a case report. [file 12905_2019_833_MOESM1_ESM.pdf]

# CARE Checklist (2013) of information to include when writing a case report

CARE case report guidelines

(cc) BY-NC-ND

| Topic | Item | Checklist item description | Reported on Page |
|---|---|---|---|
| Title | 1 | The words "case report" should be in the title along with the area of focus | 1 |
| Key Words | 2 | 2 to 5 key words that identify areas covered in this case report. | 2 |
| Abstract | 3a | Introduction—What is unique about this case? What does it add to the medical literature? | 2 |
| | 3b | The main symptoms of the patient and the important clinical findings | 2 |
| | 3c | The main diagnoses, therapeutics interventions, and outcomes | 2 |
| | 3d | Conclusion—What are the main "take-away" lessons from this case? | 2 |
| Introduction | 4 | One or two paragraphs summarizing why this case is unique with references | 3 |
| Patient Information | 5a | De-identified demographic information and other patient specific information | 3 |
| | 5b | Main concerns and symptoms of the patient | 3 - 6 |
| | 5c | Medical, family, and psychosocial history including relevant genetic information (also see timeline). | 3 - 6 |
| | 5d | Relevant past interventions and their outcomes | 3 - 6 |
| Clinical Findings | 6 | Describe the relevant physical examination (PE) and other significant clinical findings. | 3 - 6 |
| Timeline | 7 | Important information from the patient's history organized as a timeline | 6 |
| Diagnostic Assessment | 8a | Diagnostic methods (such as PE, laboratory testing, imaging, surveys). | 6 - 7 |
| | 8b | Diagnostic challenges (such as access, financial, or cultural) | 6 - 7 |
| | 8c | Diagnostic reasoning including other diagnoses considered | 6 - 7 |
| | 8d | Prognostic characteristics (such as staging in oncology) where applicable | |
| Therapeutic Intervention | 9a | Types of intervention (such as pharmacologic, surgical, preventive, self-care) | 7 |
| | 9b | Administration of intervention (such as dosage, strength, duration) | 7 |
| | 9c | Changes in intervention (with rationale) | 7 |
| Follow-up and Outcomes | 10a | Clinician and patient-assessed outcomes (when appropriate) | 7 - 9 |
| | 10b | Important follow-up diagnostic and other test results | 7 - 9 |
| | 10c | Intervention adherence and tolerability (How was this assessed?) | 7 - 9 |
| | 10d | Adverse and unanticipated events | 7 - 9 |
| Discussion | 11a | Discussion of the strengths and limitations in your approach to this case | 10 - 11 |
| | 11b | Discussion of the relevant medical literature. | 9 - 10 |
| | 11c | The rationale for conclusions (including assessment of possible causes) | 11 |
| | 11d | The primary "take-away" lessons of this case report | 11 |
| Patient Perspective | 12 | When appropriate the patient should share their perspective on the treatments they received | YES |
| Informed Consent | 13 | Did the patient give informed consent? Please provide if requested | Yes ☒ No ☐ |
